# P-1130. Association Between Procalcitonin, CRP Values and Antibiotic Use in Children with Community-Acquired Pneumonia

**DOI:** 10.1093/ofid/ofae631.1317

**Published:** 2025-01-29

**Authors:** Liliana Arriola Montenegro, Madhuri S Mulekar, Linda Hassouneh

**Affiliations:** University of South Alabama, Mobile, Alabama; University of South Alabama, Mobile, Alabama; University of South Alabama, Mobile, Alabama

## Abstract

**Background:**

Procalcitonin (PCT) and C-reactive protein (CRP) are biomarkers used to help identify bacterial etiologies of community-acquired pneumonia (CAP) as it is often challenging to differentiate between viral and bacterial causes. This study evaluated the association between PCT, CRP values, and antibiotic initiation in children with CAP.

**Figure:**
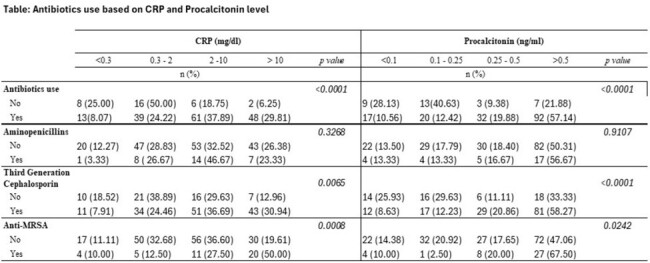

**Methods:**

This single-center, retrospective study evaluated patients aged 6 weeks to 19 years who presented with acute signs or symptoms of lower respiratory tract illness from December 1 2022 to December 31, 2023. Enrolled were patients with PCT and CRP values obtained within the first 24 hours of their hospital visit. The primary outcome was the initiation of antibiotics within the first 36 hours of the encounter. Patients with tracheostomy, prior antibiotic use, aspiration, or hospital-acquired pneumonia were excluded. PCT and CRP were categorized into 4 groups correlating to values suggesting very unlikely, unlikely, likely, and very likely bacterial infection. Bivariate and logistic regression analyses were conducted.

Patients outcomes (admission to PICU, pleural effusion and need for intubation) by Procalcitonin levels

**Figure:**
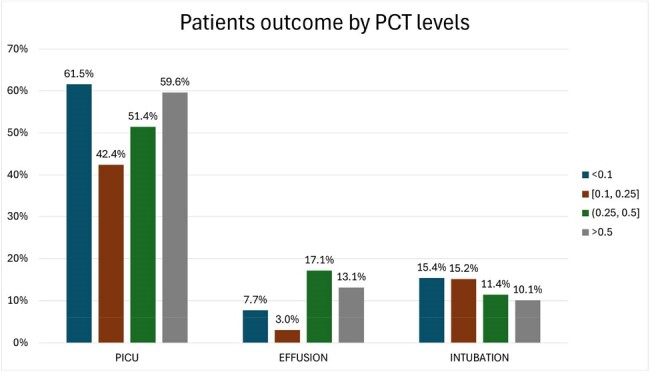

No association was seen between patient outcomes and procalcitonin levels.

**Results:**

The study included 193 patients, 83% of whom received antibiotics. Among these, 11% had a PCT < 0.1 ng/ml and 8% had a CRP < 0.3 mg/dl. Third-generation cephalosporins (3GC) were the most commonly used antibiotic (n=139, 72%). PCT and CRP values were strongly associated with initiation of antibiotics (p< 0.0001). CRP >10 mg/dl had a 14.8% increase in odds of receiving antibiotics (95% confidence interval [CI]: 2.8, 78.2), and PCT >0.5 ng/mL had a 6.9% increase in odds (95%CI: 2.3, 21.2) compared to the reference category (PCT < 0.3 ng/mL and CRP < 0.3 mg/dL respectively). A CRP of ≥10mg/dL and PCT of >0.5 ng/mL were associated with using 3GC and methicillin-resistant Staphylococcus aureus (MRSA) coverage. Days of hospitalization, pleural effusion, intubation, and PICU admission were not associated with PCT and CRP values.

**Figure:**
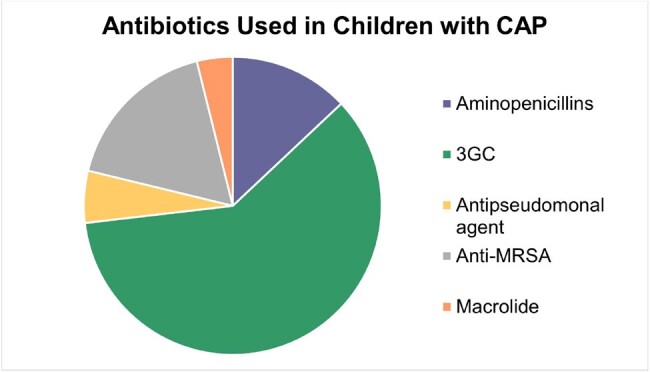

**Conclusion:**

Most subjects received antibiotics including those with normal PCT and CRP levels, suggesting a possible overuse of antibiotics. Higher levels of PCT and CRP were associated with antibiotic initiation and usage of broad-spectrum antibiotics, identifying opportunities for antibiotic stewardship.

**Disclosures:**

**All Authors**: No reported disclosures

